# *Taugt17b1* Overexpression in *Trichoderma atroviride* Enhances Its Ability to Colonize Roots and Induce Systemic Defense of Plants

**DOI:** 10.3390/pathogens12020264

**Published:** 2023-02-06

**Authors:** Shengqi Chi, Xiaoyu Xue, Ronghuan Zhang, Li Zhang, Jinfeng Yu

**Affiliations:** 1 Key Laboratory of Integrated Crop Pest Management of Shandong Province, College of Plant Health and Medicine, Qingdao Agricultural University, Qingdao 266109, China; 2 Key Laboratory of Agricultural Microbiology, College of Plant Protection, Shandong Agricultural University, Tai’an 271018, China

**Keywords:** *Trichoderma atroviride*, Glycosyltransferase Taugt17b1root colonization, plant-induced defense

## Abstract

*Trichoderma atroviride*, a soil fungus, has important applications in the biocontrol of plant diseases. Glycosyltransferases enhance the root colonization ability of *Trichoderma* spp. This study aimed to functionally characterize glycosyltransferase Taugt17b1 in *T. atroviride*. We investigated the effect of *Taugt17b1* overexpression in *T. atroviride* H18-1-1 on its biocontrol properties, especially its ability to colonize roots. Our results demonstrated that the overexpression of the *Taugt17b1* increases the *T. atroviride* colony growth rate, improves its root colonization ability, promotes the growth and activity of the defensive enzymatic system of plants, and prevents plant diseases. This study put forth a new role of *T. atroviride* glycosyltransferase and furthered the understanding of the mechanisms by which fungal biocontrol agents exert their effect.

## 1. Introduction

Fungi of the genus *Trichoderma* are outstanding among filamentous ascomycetes because of their high adaptability to various ecological conditions and lifestyles [1]. These fungi are well known for their ability to exert beneficial effects on plants, including the promotion of growth and the induction of resistance to disease [2]. They can produce a wide range of antibiotics, e.g., peptaibols and terpenoids with broad-spectrum action, as well as fungal cell-wall-degrading enzymes (CWDEs) [3,4,5]. The variety and amount of these compounds produced depend on the strain. Gliotoxin was the first *Trichoderma*-derived compound with antifungal activity; thus, an antagonistic role was postulated for *Trichoderma virens,* from which it was discovered [6]. The biocontrol mechanisms of *Trichoderma* spp. include direct (such as mycoparasitism and antibiosis) and indirect attacks (for example, competing for space and nutrients), increasing plant growth and development, and inducing plants’ systemic resistance to pathogens on plant pathogenic fungi [7].

Fungi belonging to the genus *Trichoderma*, commonly found in soil or colonizing plant roots, exert beneficial effects on plants, including the promotion of growth and the induction of resistance to disease [8].

So far, many *Trichoderma* spp. have been extensively researched and exploited by scientists worldwide, especially targeted at the direct effect of *Trichoderma* fungi on pathogens, but indirect effect is still a poorly researched mechanism.

In addition to the ability of *Trichoderma* spp. to directly attack the growth of pathogens, evidence suggests that they can enhance root growth and development, crop productivity, resistance to abiotic stresses, and the uptake and use of nutrients in the outer cells of plant roots (their infection is limited to these outer layers) [5]. Furthermore, an increasing number of studies have focused on the molecular features underlying the *Trichoderma*–plant–pathogen interaction, which is an indirect and intricate process involving communication between molecules generated by *Trichoderma* spp. and plants [8]. The signaling molecules secreted by *Trichoderma* spp. can activate the expression of systemic resistance-related genes in plants, which protects them against pathogens [9]. Crosstalk between a complex network of signal transduction pathways, involving salicylic acid (SA), jasmonic acid (JA), reactive oxygen species (ROS), and ethylene (ET), regulates plant defense against pathogens [10]. In addition, pathogenesis-related (PR) genes, a series of marker genes related to SA, JA, and ET signaling, are also involved in these defense transduction pathways [11]. Considerable attention has been paid to the induction of resistance, which protects plants from diseases [12,13,14]. The ability to antagonize, parasitize, or kill other fungal pathogens—which forms the crux of *Trichoderma*–pathogenic microorganism interactions—seems to be widespread among *Trichoderma* species.

Fungi communicate and perceive their environment through peptides that they secrete [15]. The sucrose in root exudates attracts fungus and is essential for *Trichoderma*–root interactions. While hydrophobin-like proteins mediate its root attachment, the secreted swollenins and plant cell-wall-degrading enzymes facilitate the internalization of the fungal hyphae [16,17]. Certain components of the *Trichoderma* spp. secretome induces susceptibility in host plants, perhaps by suppressing initial plant resistance, which aids fungal root penetration [18]. Bioinformatic predictions suggest that some secreted *Trichoderma* spp. proteins could have plant intracellular targets [19]. This could be a promising direction for future experiments.

Glycosyltransferases (GTs) are enzymes involved in the biosynthesis of oligosaccharides, polysaccharides, and glyco-conjugates [20]. These molecules have enormously diverse functions, including those related to structure, storage, and signaling [21]. In eukaryotes, most glycosylation reactions occur in the Golgi apparatus. Golgi-resident GTs are type II transmembrane proteins with a large C-terminal globular catalytic domain facing the luminal side [21]. Interestingly, two soluble secreted GTs were identified in the mycoparasitic fungi *T. atroviride* and *T. virens* (one in each) [22]. These two extracellular GTs are expected to regulate the biocontrol properties of the two *Trichoderma* species.

In this work, we investigated the effect of *Taugt17b1* overexpression in *T. atroviride* H18-1-1 on its biocontrol properties, especially the ability to colonize roots. Our results demonstrated that an overexpression of the Taugt17b1 increases *T. atroviride* colony growth rate, improves its root colonization ability, promotes the growth and activity of defensive enzymatic systems in plants, and prevents plant diseases, thus enhancing the biocontrol properties of *T. atroviride* H18-1-1.

## 2. Materials and Methods

### 2.1. Strains and Culture Conditions

*T. atroviride* H18-1-1 (wild type strain-WT), a highly effective antagonistic strain, which has been stored in the General Microbiology Center of the China Microbiological Culture Preservation and Management Committee: CGMCC No. 9774., was previously screened in our laboratory. Overexpressing transformants used throughout this study were routinely maintained on potato dextrose agar (PDA) at 28 ℃, unless otherwise indicated. The number of conidia produced by each strain were counted under microscope with a hemacytometer (cat: YA0810, Beijing Solebo Technology Co., Ltd, Beijing, China). All bacterial cultures were maintained on Luria–Bertani agar (LBA) at 37 °C, and liquid cultures were grown with shaking at 180 rpm.

### 2.2. Identification, Characterization and Acquisition of Overexpression Strains

The selected *Taugt17b1* gene encodes the beta-1,4-mannosylglycoprotein 4-beta-N-acetylglucosaminyltransferase protein in *T. atroviride. Taugt17b1* was identified and characterized using the nucleotide and amino acid sequences obtained from NCBI. The Pfam v32.0 database was used to identify the protein family. The programs SignalP 4.1 and transmembrane hidden Markov models (TMHMM 2.0, SIB Swiss Institute of Bioinformatics, Lausanne, Switzerland) were used for the prediction of cell secretion signals and transmembrane helices, respectively. A phylogenetic tree was generated based on the neighbor-joining method using MEGA version 7.0. The numbers at the nodes represent the percentage of their occurrence in 1000 bootstrap replicates. The above-mentioned tools can be found at the Center for Biological Sequence Analysis (http://www.cbs.dtu.dk/biotools/, accessed on 4 October 2022).

To overexpress the *Taugt17b1*, we used the expression vector pFY11, which has the selection marker *Npt* II (neomycin phosphotransferase II, which confers resistance to the antibiotic geneticin) under the strong constitutive promoter RP27 (derived from the *Magnaporthe grisea* ribosomal protein 27 gene). We amplified the *Taugt17b1* ORF with the primer pair OEGTF/OEGTR (Table S1), using Phanta Max Super-Fidelity DNA Polymerase (Vazyme Biotech Co, Jiangsu, China CAT: P505-d1) and cloned it into pYF11, using the *Xho*I linearized vector and the ClonExpress Ultra One Step Cloning Kit (Vazyme Biotech Co, Jiangsu, China CAT: C115-01), in accordance with the manufacturer’s instructions. The recombinant plasmids (a total of 20 μg) were introduced into the *T. atroviride* H18-1-1 fungal protoplasts using polyethylene glycol-mediated fungal transformation, and the resulting transformants were selected on TB3 (3 g yeast extract, 3 g casamino acids, 200 g sucrose, 9 g agar in 1000 mL distilled H_2_O) medium containing G418 (Geneticin) and identified by a polymerase chain reaction (PCR) with primers (JCGTF/JCGTR). To assess the sensitivity of *T. atroviride* H18-1-1 to G418, different concentrations of G418 (0, 10, 15, 20, 25, and 30 mg/mL) were added to PDA medium and incubated at 28 °C. Growth was observed every 24 h. Experiments were conducted in triplicates for each treatment group.

The genetic stability of the verified positive transformants was determined by culturing them in PDA medium with G418 for 10 generations. Conidia were harvested with sterile distilled water and inoculated on PDA medium containing G418. The loss of plasmids was crosschecked by analyzing the DNA and RNA of the strains. The relative transcription levels of the *Taugt17b1* gene were assessed via qRT-PCR analysis to detect the genetic stability of the transformants. We named the transformed overexpression strain as H18-1-1-t.

### 2.3. In Vitro Mycoparasite Effect of Trichoderma Mutants on Growth Plant Pathogenic Fungi

The *T. atroviride*, and plant pathogenic fungi (*Fusarium proliferatum*, *F. oxysporum*, *F. verticillioides*, *F. graminearum*, *F. pseudograminearum*, *Gaeumannomyces graminis*, *Rhizoctonia cerealis,* and *Bipolaris sorokiniana*) were grown on PDA, and 5 mm plugs were removed from the actively growing colony edge for confrontation experiments. Plugs from the *T. atroviride* were placed on one edge of a fresh PDA plate. Each strain was confronted with a plug of a competing fungus, and incubated for 72 h. Experiments were completed in triplicates and growth characteristics and inhibition were measured every 24 h.

### 2.4. Determining Root Colonization Ability of T. atroviride H18-1-1 WT and H18-1-1-t Strains and Root Activity

The root colonizing abilities of the H18-1-1 WT and H18-1-1-t strains were compared using qRT-PCR analysis. Briefly, surface-disinfected wheat (Lumai 21) seeds were planted in a culture tube containing fine sand. The sterile sand mix (170 g) was drenched with 5 mL of water containing H18-1-1 WT and H18-1-1-t conidia (2 × 10^5^ conidia/mL). After 3 days of growth, the roots were harvested, rinsed with distilled water, and ground in liquid nitrogen. The root activity of the wheat was determined using the 2,3,5-triphenyltetrazolium chloride (TTC) method. The relative colonization capacities of the H18-1-1 WT and H18-1-1-t strains were assessed by performing qRT-PCR of the genetic material isolated from the roots. *Actin* and *Pal* (phenylalanine ammonia lyase ) were used as internal standards [23]. The primers used for them are listed in Table 1 (ACTINF/ACTINR, PLAF/PLAR). Experiments were carried out in triplicates for each strain.

### 2.5. Effect of T. atroviride H18-1-1-t on Plant Defensive Enzymatic System

Here, wheat roots were used to determine the effects of *Taugt17b1* overexpression in H18-1-1-t on plant defense enzymes. The conidia of H18-1-1 WT and H18-1-1-t strains were harvested and resuspended in sterile distilled water (10^5^ conidia/mL). Subsequently, the wheat seeds grown in sterile sand (3 kg) containing *Rhizoctonia cerealis* (sharp eyespot disease) were treated with 100 mL each of these two conidial suspensions. Water was used in the blank control group. The results were observed after 30 d. The wheat leaf defense enzymes’ catalase (CAT), peroxidase (POD), and superoxide dismutase (SOD) activities were estimated using a POD, CAT, and SOD activity detection kit (cat: BC0090, BC0205, BC0170; Solarbio Science & Technology Co., Ltd., Beijing, China), according to the manufacturer’s instructions. Experiments were carried out in triplicates for each treatment group.

The effect of *Taugt17b1* overexpression on plant defense enzyme gene expressions were studied using Pak Choi. Undamaged Pak Choi seeds of similar sizes were planted in sterile soil in a flowerpot. After germination, spore suspension of H18-1-1 WT and H18-1-1-t was added at a concentration of 10^5^ conidia/mL respectively. Different treatment protocols were used. The control group (CK) was applied with 50 mL of sterile water. A total of ten Pak Choi samples were randomly selected for qRT-PCR analysis. Tests for every treatment group (3 sets each) were done in triplicates.

### 2.6. Effect of T. atroviride H18-1-1 WT on Disease Resistance in Wheat

Wheat seeds were sown in sterilized soil and inoculated with *Rhizoctonia cerealis* 21 days after germination. In the treated groups, 100 mL spore suspension of the H18-1-1 WT and H18-1-1-t strains were added to each pot at a concentration of 2 × 10^5^ conidia/mL and covered with a 1 cm thick mixture of matrix and soil. The extent of infection with wheat sharp eyespot was measured one week later [24].

### 2.7. Quantitative Reverse Transcription Polymerase Chain Reaction (qRT-PCR) Assays

The total RNA was extracted using an EasyPure RNA Kit (cat: ER101-01, TransGen Biotech, Beijing, China), according to the manufacturer’s instructions. qRT-PCR experiments were carried out according to the manufacturer’s instructions (cat: Q221-01, Vazyme Biotech Co, Jiangsu, China). The relative transcription levels were measured using the 2^−ΔΔCT^ method with *Actin* as the reference [25]. All primers used for the qRT-PCR assays are listed in Table S1.

### 2.8. Statistical Analysis

The data are presented as mean ± standard error values. The differences among variables were analyzed using Duncan’s multiple range test or Student’s t-test with significance set at *p* < 0.05.

## 3. Results

### 3.1. Results and Analysis

#### 3.1.1. Identification of the *Taugt17b1*

**Figure 1:**
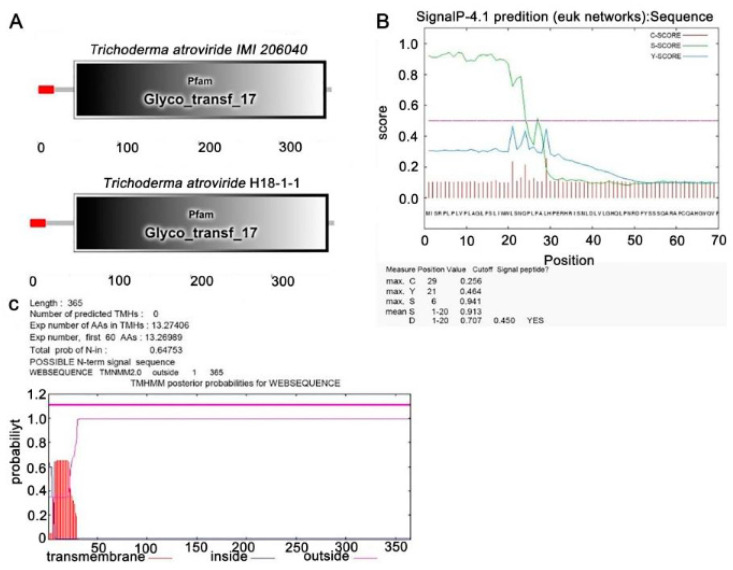
Bioinformatics analysis of *Trichoderma atroviride* H18-1-1 *Taugt17b1.* (**A**) Analysis of SMART domain of *T. atroviride* H18-1-1 Taugt17b1 and *T. atroviride* IMI 206040 Taugt17b1; (**B**) signal peptides sequence prediction of *T. atroviride* H18-1-1 Taugt17b1; and (**C**) prediction of transmembrane helices of *T. atroviride* H18-1-1 Taugt17b1.

The *Taugt17b1* gene in *T. atroviride* was identified using Blastn (nucleotide BLAST) from GenBank (XM_014090373) [26]. SMART analysis revealed that *Taugt17b1* is predicted to encode a 365-amino-acid-long protein. It contains a Glyco_transf_17 domain (ID 25780088) (Figure 1a). In addition, the *Taugt17b1* protein contains a cell secretion signal and has no predicted transmembrane helices in *T. atroviride* H18-1-1, (Figure 1b,c). A phylogenetic tree of Taugt17b1 proteins from *Trichoderma atroviride* and other fungi indicates that these Taugt17b1 proteins were divided into one clade (Figure S1). These results indicate that *Taugt17b1* is secreted via the glycosyltransferase protein.

#### 3.1.2. *Taugt17b1* Overexpression Obtained Was Stably Inherited

G418 was used as a resistance screening marker for *T. atroviride* H18-1-1. Since G418 has never been used as a resistance screening marker for *T. atroviride* before, we had to test the sensitivity of *T. atroviride* H18-1-1 to G418. H18-1-1 could not grow on PDA medium supplemented with 25 μg/mL of G418, even after 96 h of incubation. The results indicate that the optimal concentration of G418 needed for screening H18-1-1-t is 25 μg/mL (Table 1).

**Figure 2:**
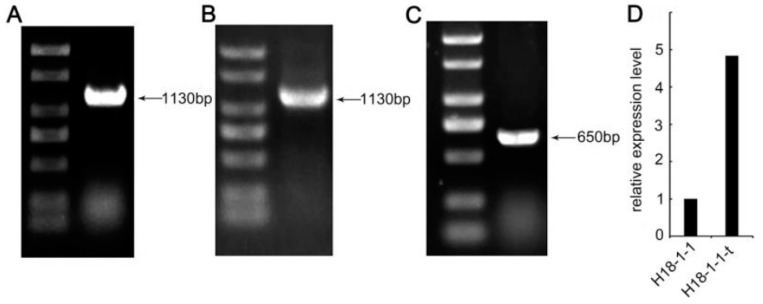
The confirmation of *Taugt17b1* gene overexpression in *T. atroviride* H18-1-1 (H18-1-1-t) strain, (**A**) PCR detection of yeast protoplast transformation; (**B**) PCR detection of *Escherichia coli* transformation; (**C**) PCR detection of protoplast transformation of *T. atroviride*; and (**D**) the relative expression level of *taugt17b1* gene.

The *Taugt17b1* overexpression vector was generated and transformed into protoplasts of the *T. atroviride* H18-1-1 strain using the PEG-mediated method [27]. The transformants were selected using PDA plates containing 25 μg/mL of G418 and further verified using PCR (Figure 2a,b,c). A total of 13 transformants were obtained and all transformants were verified as positive. In addition, the expression levels of *Taugt17b1* in H18-1-1 and the transformants were assessed by qRT-PCR analysis, and the results showed that the highest expression level of *Taugt17b1* was 4.8-fold higher than that in the WT strain. The strain with the highest expression level was called H18-1-1-t and was selected for subsequent experiments (Figure 2d).

**Figure 3:**
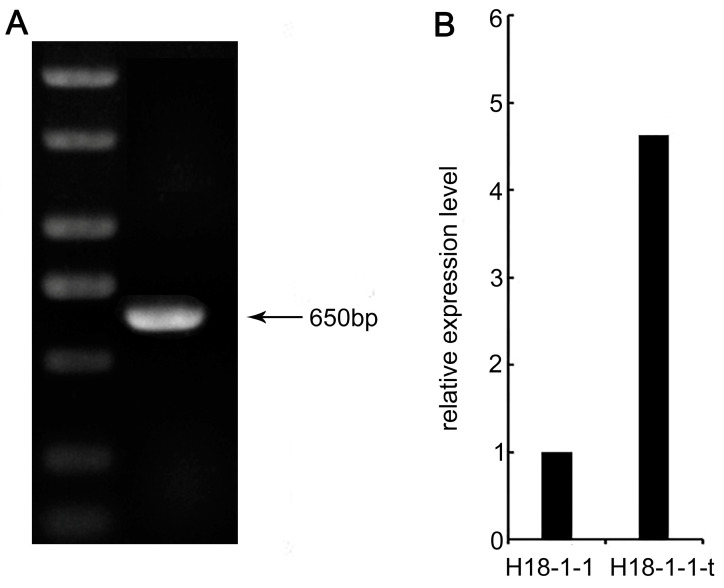
Detection of stability of the *taugt17b1* overexpression plasmid vector system in *T. atroviride* H18-1-1 strain, (**A**) the presence of *taugt17b1* gene was confirmed in overexpression strain H18-1-1-t after 10 culture passages; and (**B**) the relative expression level of *taugt17b1* gene in H18-1-1-t strain after 10 generations.

The H18-1-1-t screened by the above experiments can be considered to have research significance only if the vector is stably inherited. Therefore, we assessed the genetic stability of the transformed *Taugt17b1* overexpression vector. The pYF11–*Taugt17b1* overexpression vector plasmid was detected in H18-1-1-t even after 10 generations of passaging (Figure 3a). Furthermore, the expression level of *Taugt17b1* in H18-1-1-t was 4.62 times higher than in the H18-1-1 WT strain (Figure 3b). These results showed that the plasmid *Taugt17b1* overexpression vector, transferred into the *T. atroviride* strain H18-1-1, can be stably inherited, and the ability to overexpress the *Taugt17b1* remains unchanged over generations.

#### 3.1.3. *Taugt17b1* Overexpression Has No Effect on the Growth and Conidiation of *T. atroviride*

**Figure 4:**
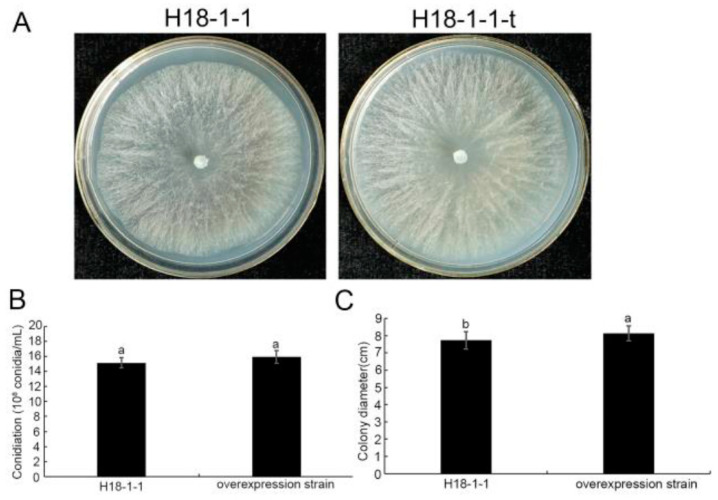
Growth and conidiation of *T. atroviride* H18-1-1 WT and H18-1-1-t strains, (**A**) growth of H18-1-1 WT and H18-1-1-t strains on potato dextrose agar (PDA); (**B**) number of conidia in PDA cultures were examined after incubating for 7 days; and (**C**) colony diameter of H18-1-1 WT and H18-1-1-t strains measured in PDA medium, incubated at 28 ℃ for 28 h. Different letters on the bars for each treatment indicate significant difference at *p* < 0.05 with Duncan’ s multiple range test. All experiments done in triplicates.

The growth rates of H18-1-1 and H18-1-1-t were measured. The results showed that the colony growth rate of the H18-1-1-t strain was accelerated, however there was no significant difference between H18-1-1 and H18-1-1-t in their morphology and color (Figure 4a,c). The conidia produced by each strain were counted under microscope; results indicated that there was no significant difference between H18-1-1-t and H18-1-1 in the conidiation (Figure 4b).

#### 3.1.4. Antagonistic Effects of Strains H18-1-1 and H18-1-1-t on Phytopathogenic Fungi

**Figure 5:**
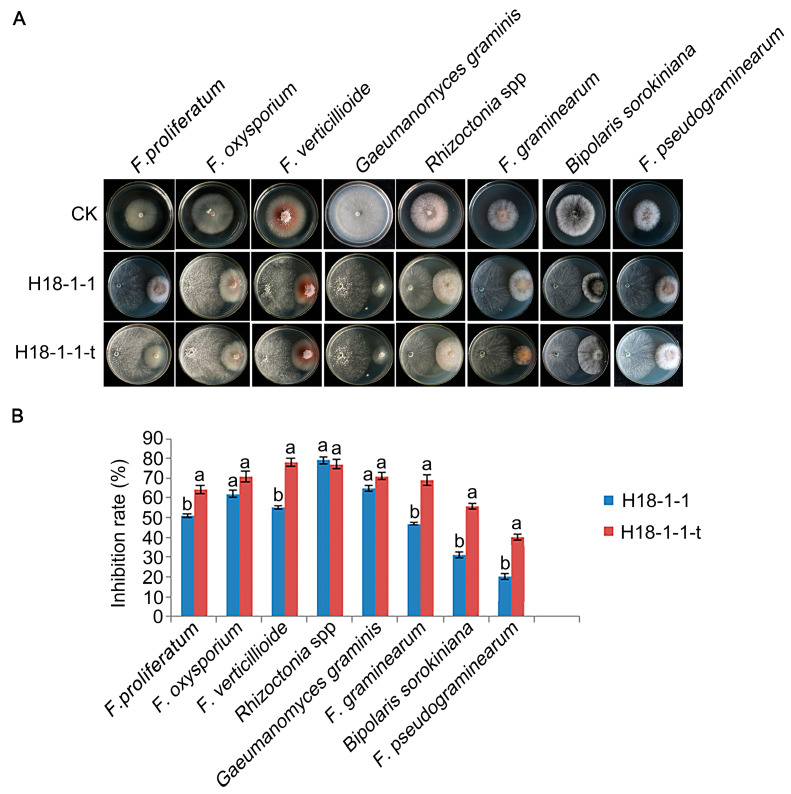
The inhibition of plant pathogenic fungi in plants treated with H18-1-1-t strain. (**A**) Confrontation of H18-1-1 WT and H18-1-1-t strains with various plant pathogenic fungi; and (**B**) relative inhibition rates of H18-1-1 WT and H18-1-1-t strains on various plant pathogenic fungi. Different letters on the bars for each treatment indicate significant difference at *p* < 0.05 with Duncan’ s multiple range test. All experiments were done in triplicates.

To confirm whether the overexpression of *Taugt17b1* in *T. atroviride* would improve antagonism against phytopathogenic fungi, we confronted the H18-1-1-t strain with eight pathogenic fungi roots. The strains H18-1-1 WT, H18-1-1-t, and plant pathogenic fungi were grown on PDA for 48 h. We found that the antagonistic effect of H18-1-1-t on phytopathogenic fungi studied here was increased, except in *Rhizoctonia* spp., in comparison with that of the H18-1-1 WT. The antagonism was stronger against *F. pseudograminearum*, *F. verticillioides*, *F. graminearum*, *F. oxysporum, F*. *proliferatum, Bipolaris sorokiniana*, and *Gaeumannomyces graminis* (Figure 5).

#### 3.1.5. Overexpression of *Taugt17b1* Affects Colonization Ability of H18-1-1 and Root Activity

**Figure 6:**
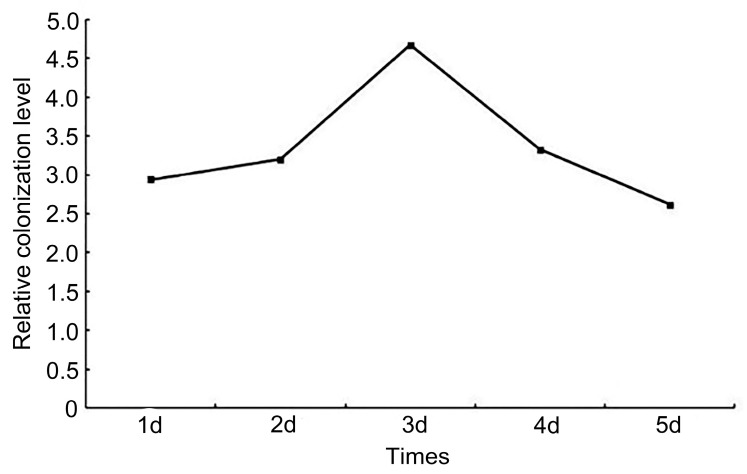
Colonization ability of the overexpression H18-1-1-t strain.

We assessed the ability of the root colonization of the H18-1-1-t and H18-1-1 WT strains using qRT-PCR. The colonization ability of the H18-1-1-t was higher than that of the H18-1-1 WT strain; it peaked on day 3 (Figure 6). The results showed that the overexpression of the *Taugt17b1* gene in *T. atroviride* significantly increased their colonization ability.

The root activity of the wheat was tested using the TTC method. The results showed that the root activity of the wheat treatment with H18-1-1-t is higher than in the case of treatment with H18-1-1 (Table 2). These results indicate that the *Taugt17b1* overexpression can enhance the ability of *T. atroviride* to affect the root activity of wheat.

#### 3.1.6. Overexpression of the *Taugt17b1* Improved Activity and Expression of Plant Defense Enzymes

**Figure 7:**
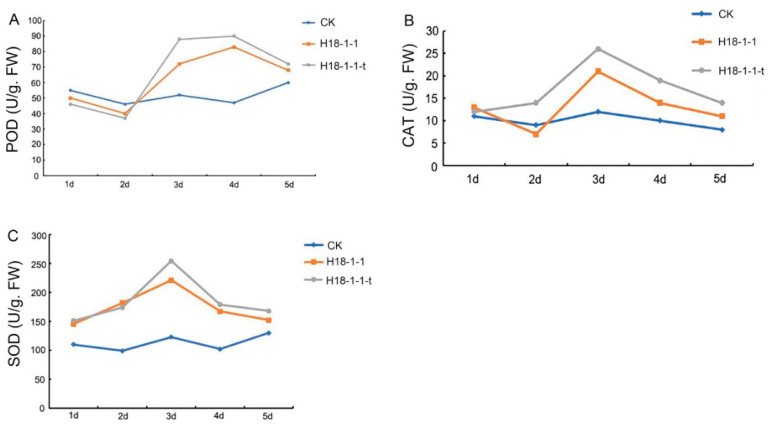
The influence of *T. atroviride* H18-1-1 WT and H18-1-1-t strains on the enzyme activities. (**A**) The influence on the *POD* enzyme activity; (**B**) The influence on the *CAT* enzyme activity; (**C**) The influence on the *SOD* enzyme activity.

**Figure 8:**
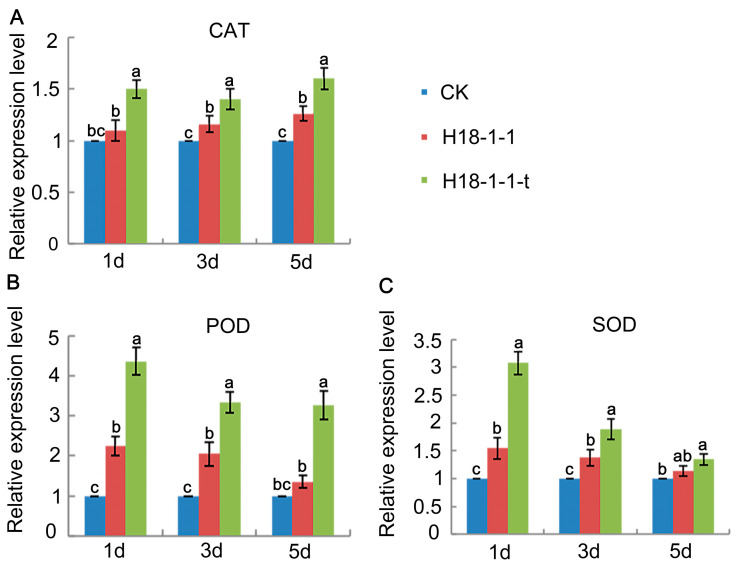
Effect of *Trichoderma* treatment on relative expression of plant defense enzyme genes in Chinese cabbage, Pak Choi, (**A**) expression level of *CAT* gene; (**B**) expression level of *POD* gene; and (**C**) expression level of *SOD* gene.

To investigate the effect of *Taugt17b1* overexpression on the defense enzyme activity, we examined the POD, CAT, and SOD activity of the wheat treated with H18-1-1-t and H18-1-1. The results showed that the enzyme activities (SOD, POD, and CAT) of wheat plants treated with H18-1-1-t and H18-1-1 were higher than those treated with water (CK). In addition, the enzyme activities of the wheat plants treated with H18-1-1-t were higher than those treated with H18-1-1 (Figure 7b,c). Furthermore, the expression levels of the plant defense enzyme coding genes—POD, SOD, and CAT—in Pak Choi (*Brassica chinensis*) were determined by greenhouse and field experiments. The increase in their gene expression caused by the treatment with the H18-1-1 WT and H18-1-1-t strains peaked on the first day. *POD* expression was increased in the Pak Choi treated with both H18-1-1 WT and H18-1-1-t, as compared to the control group. However, H18-1-1-t caused a greater increase in *POD* expression than H18-1-1 WT (Figure 8).

#### 3.1.7. The Control Effects of H18-1-1-t in Wheat Sharp Eyespot Was Better Than H18-1-1

H18-1-1-t controlled the wheat sharp eyespot spread much better, by approx. 12.6 %, more than the H18-1-1 WT strain. The disease index in the wheat treated with H18-1-1-t was 19.1% lower when compared to the wheat treated with the H18-1-1 WT strain (Table 2). These results showed that H18-1-1-t had a better control effect on the wheat sharp eyespot than the WT strain.

## 4. Discussion

The biocontrol mechanism of *Trichoderma* against plant diseases is a current research hotspot in the field of biological control [28]. Biocontrol imparted by *Trichoderma* spp. would involve mechanisms such as competition with pathogenic fungi for nutrition and space, parasitism of pathogenic fungi, and the production of antibiotics to inhibit the growth and reproduction of pathogenic fungi, as well as the promotion of plant growth and the activation of disease resistance systems in plants, thereby indirectly controlling the infection of plants with pathogenic fungi [29]. By controlling fungal diseases, *Trichoderma* spp. can improve the plant biomass and germination rate, plant disease resistance and stress resistance, and the soil microbial community structure.

Atanasova et al. [30] found that *sfp2* overexpression in *T. atroviride* can enhance the mycelial growth rate of *T. virens* and its antagonistic ability against pathogens. Overexpression of the *T***.** *guizhouense* gene increased the growth-promoting effect of *T. guizhouense* on cucumber [31]. Montero-Barrientos [32] overexpressed the *HSP70* gene in the *T. harzianum* T34 strain and found that it could improve the resistance of the strain to heat and abiotic stress.

The colonization of plant roots by *Trichoderma* spp. is regulated by salicylic acid and other signals. *Trichoderma* with certain biocontrol effects must have an excellent colonization ability. Jorge [33] isolated and identified *Thkel1* from *T. harzianum* and used qRT-PCR to detect its colonization ability, finding that *Thkel1* was related to the colonization ability of *T. harzianum* in Cruciferae plants. Guzmán-Guzmán [34] discovered that the overexpression of the hydrophobic protein TvhydⅡ in *T. virens* can improve both the antagonistic activity of *T. virens* against *R. solani* AG2 and its root colonization ability. Glycosyltransferase is the key enzyme in the glycosylation reaction, which plays a very important role in the production of antibiotics. Many glycoside antibiotics have undergone stereoselective and regioselective glycosylation in their synthesis, which can enable antibiotics to specifically recognize biological targets and improve the antibacterial activity of antibiotics. In this study, qRT-PCR was used to detect the colonization ability of *Trichoderma* spp. in wheat roots, and it was found that the overexpression of *Taugt17b1* significantly improved the colonization ability of *T. atroviride*.

*Trichoderma* spp. can induce the expression of defensive enzymes in host plants and increase their activity, thereby improving the disease resistance in plants and reducing the occurrence of diseases. Zhang found that *T. harzianum* reduced the adverse effects of *F. oxysporum* on soybean. An analysis of reactive oxygen species’ (ROS) scavenging enzyme activity showed that the activities of POD and SOD increased significantly in *Trichoderma*-pretreated plants [35]. Here, using the greenhouse pot experiment, we found that in wheat, the *Taugt17b1-*overexpressing H18-1-1-t strain increased the activity of the three plant defense enzymes (SOD, POD, and CAT) to a greater extent than the H18-1-1 WT strain. This indicated that the *Taugt17b1* overexpression in *Trichoderma* spp. could improve the defense enzyme activity of wheat.

*Trichoderma* spp. have a significant inhibitory effect on common diseases in crops [36,37]. The overexpression of the aquaglyceroporin gene in *T. harzianum* enhances its antagonism activity [38]. The overexpression of the *chit42* gene in *T. harzianum* enhances its antagonistic activity against *Botrytis cinereal* [39]. In this study, the incidence of wheat sharp eyespot was significantly reduced in the wheat treated with spore suspensions of the *Taugt17b1-*overexpressing *T. atroviride* strain (H18-1-1-t), compared to those treated with the wild-type strain (H18-1-1 WT). At the same time, the plate confrontation experiment showed that the overexpression of *Taugt17b1* increased the ability of *T. atroviride* to inhibit the plant pathogens.

In summary, this study shows that the overexpression of *Taugt17b1* can increase the *T. atroviride* colony growth rate and improve the *T. atroviride* colonization in plant roots. H18-1-1 exerts biocontrol effects in terms of pathogen antagonism and growth promotion.

## Figures and Tables

**Table 1:** Sensitivity test of *Trichoderma atroviride* H18-1-1 to G418.

G418 (μg/mL)	0	10	15	20	25	30
H18-1-1	+	+	+	+	-	-

+ positive; - negative.

**Table 2:** Disease index, disease prevention and root vigor in *T. atroviride* H18-1-1 WT and H18-1-1-t strains.

Treatment	Disease Index	Disease Prevention	Root Vigor (μg/g/h)
CK	38.33a	0	176.45b
H18-1-1	16.47b	57.03	335.64a
Overexpression strain	13.33b	65.22	389.87a

## Data Availability

Not applicable.

## Supplementary Materials

The following supporting information can be downloaded at: https://www.mdpi.com/article/10.3390/pathogens12020264/s1, Table S1: primers used in this study. Figure S1. Phylogenetic analysis of Taugt17b1 in Trichoderma atroviride and other fungi. A phylogenetic tree was generated based on the neighbor-joining method using MEGA version 6.0. The numbers at nodes represent the percentage of their occurrence in 1000 bootstrap replicates.
