# Supplementary material for: Taugt17b1 Overexpression in Trichoderma atroviride Enhances Its Ability to Colonize Roots and Induce Systemic Defense of Plants
Source: Pathogens. 2023 Feb 6;12(2):264. doi: 10.3390/pathogens12020264 (PMC9959489; doi:10.3390/pathogens12020264)
Supplement: Supplementary file 1 [file pathogens-12-00264-s001.zip › pathogens-2138862-supplementary.pdf]

Table S1 Primers used in this study.

Primer	Sequence
GTAF	TGTTGCTGGTTTGGTTGCTTGTT
GTAR	TGCGATGTCGTTCGGGATG
JCGTF	TCACAGCCCGTCTTCAGAACACTTTGC
JCGTR	CACAGCCCGTCTTCAGAACACTTTGC
OEGTF	ACTCACTATAGGGCGAATTGGGTACTCAAATTGGTTTGATCTCGCGGCCACTTCC
OEGTR	CACCACCGGTGAACAGCTCCTCGCCCTTGCTCACAGCCCGTCTTCAGAACACTTTGC
PLAF	GAGGTCTTGTCTGCGGTGTTCTG
PLAR	CGGTCCTGCTTCGGCTTCATC
QRTGTF	CAACAAGGCGCTGATTGTGC
QRTGTR	CCTGAACCACTCGGGAAAG
ACTINF	CAAGTCCTCGACGTTGTCCG
ACTINR	CCACCGAGAGAGTGGGTGAT
TaActinF	CACTGGAATGGTCAAGGCTG
TaActinR	CTCCATGTCATCCCAGTTG
G418F	AGGGGCTTCGGCGTCATC
G418R	TTGGGCGTCGTCGTGTCC

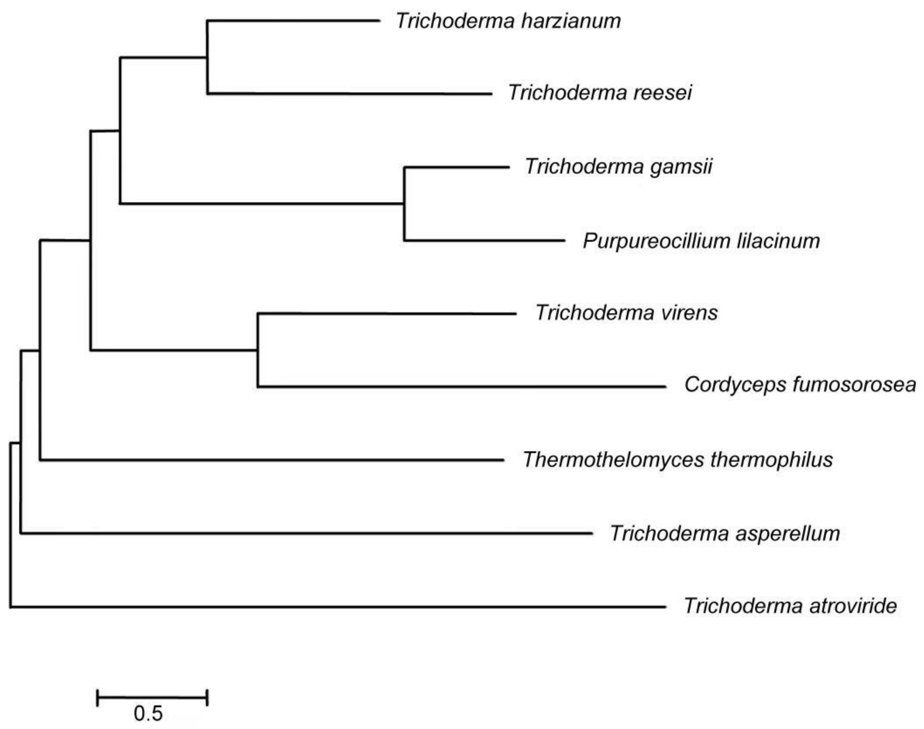

Figure S1 Phylogenetic analysis of *Taugt17b1* in *Trichoderma atroviride* and other fungi. A phylogenetic tree was generated based on the neighbor-joining method using MEGA version 6.0. The numbers at nodes represent the percentage of their occurrence in 1000 bootstrap replicates.
